# Concerning the Action of Opium upon the Uterus, and Particularly as a Parturient Agent

**Published:** 1869-07

**Authors:** P. C. Barker

**Affiliations:** Morristown, N. J.


### CONCERNING THE ACTION OF OPIUM UPON THE UTERUS, AND PARTICULARLY AS A PARTURIENT AGENT.

BY P. C. BARKER, M.D., Morristown, N. J.

"Parturition consists in the expulsion of the fœtus and its appendages from the cavity of the uterus, and ends in the separation of the child and the mother" (Churchill). It is accomplished by means of non-striated contractile fibres, which are practically muscular, and constitute what is generally termed "the muscular coat of the uterus." These fibres are arranged in longitudinal, oblique, and (circular) transverse directions. The two first predominate in the body, and the latter in the cervix, and particularly in the "os uteri externum."

Before the expulsive contractions commence, which terminate the process, these circular fibres should relax, and the "os" become widely dilated; but irregular contractions of the different sets of fibres, or contractions of the circular alone (constituting false pains), or rigidity of the "os uteri," due to tonic contraction of its circular fibres, often prevent this physiological dilatation, and prove the source of great distress to the patient and annoyance to the obstetrician.

It was in cases of this character that I discovered in opium a valuable parturient agent. I will narrate a few cases which will illustrate how it became manifest to me:—

CASE I. Mrs H., about 25, a strong and healthy woman, primipara, was taken in labor, 2 P.M., July 15th, 1862. Saw her soon after. Pains frequent, and of moderate severity; os dilated sufficiently to admit the point of the index-finger. Head presenting in first position. Left her for half an hour, and upon returning found her condition unchanged. Visited her at intervals until late in the evening, when, no progress having been made, ordered antimon. et potass.-tart., gr. $\frac{1}{8}$, every half hour. Went home, leaving directions that I should be sent for if any change occurred. Messenger came to me about 2 A.M., 16th. No further dilatation. She complained very much of the severity of the pains. Tartar emetic, gr. $\frac{1}{4}$, every half hour. No improvement resulting, and nausea being constant, it was discontinued in the morning; and a stream of warm water was thrown upon the os, by means of a Davidson's syringe, for half an hour. This was repeated three times during the morning.

*2 P.M.* Still no dilatation. Patient a little feverish, and complaining of being very tired and sleepy. Ordered morph. sulph., $\frac{1}{4}$th, and left her to get a little sleep, while I went to attend to other engagements. Returning about an hour after, I found mother and child comfortably asleep side by side. About half an hour after taking the morphine she had a hard expulsive pain; and before any one could leave the house the child was born. The pains had not changed in character until just before the termination of the labor, nor had she been asleep.

Case II. Mrs. O., 35, multipara. Previous confinement easy. Taken in labor with her fourth child, November, 1863. Pains of same character as in Case I., for 16 hours producing little dilatation. At 1 A.M., gave morph. sulph., gr. $\frac{1}{3}$, hoping to quiet the pains. Left for home, a short distance away, and retired at once. Just as I was getting asleep, a messenger came for me in hot haste, stating that the child was born. Hastily dressing, I returned in time to remove the placenta. Less than three-fourths of an hour had elapsed since I quitted the house. She had but one pain, of different character from those which had tormented her so long unavailingly, previous to taking the morphine.

Case III. Mrs. W., about 24, primipara, sent for me early in the evening. Being absent from home, my former partner, Dr. F. D. Leute, of Cold Spring, N. Y., answered the summons. Upon my return I went to relieve the Doctor. There was no dilatation, although the pains had been recurring for some time. We gave her morph. sulph., gr. $\frac{1}{4}$. and went home, giving directions to send for me should any change occur.

On the following day, I learned that about half an hour after our departure she was seized with severe expulsive pains, which terminated the labor before a messenger could be dispatched after me.

It is obvious that morphine was given in these cases, as it is usually given by the profession, if at all, with the intention of obtaining a respite from the pains, in the hope that when they returned they would be more efficient. I was surprised in each case at the result; although in the first I attributed it to the repeated use of the warm douche, from its recognized power in certain cases of this character. In the succeeding cases I was obliged to look for a different explanation, and at length concluded that the morphine increased the expulsive power of the body of the uterus to a degree sufficient to overcome the circular fibres and connecting tissue of the "os uteri." Further light was thrown upon the subject by the following case:—

CASE IV. Mrs. G., 28, multipara. In labor a number of hours. Os uteri remaining about half dilated, and rigid. Gave morph. sulph., gr. ¼. About half an hour after, while making an examination during a pain, my first and second fingers being applied to opposite sides of the os, in order that I might observe the effect of the pain upon its hitherto unyielding tissues, I was surprised to feel it easily dilating.*

In this case, I suspected that while opium stimulated the fibres of the body of the uterus (longitudinal and oblique), it also relaxed the circular fibres of the os. Further observation, in a large number of cases of varied character, has convinced me that opium, instead of having a general anodyne effect upon the uterus, possesses this special power as a parturient agent. I say general anodyne effect, for while it sometimes quites uterine contractions (witness its universal use for this purpose), yet it is in those cases in which the circular fibres are called into action alone, or where the longitudinal and oblique fibres contract irregularly—in short, in *false pains.* I am fully persuaded that *opium never did or can arrest a physiological* labor.

I have many times been called to cases in which the pains have returned regularly and with increasing intensity for a number of hours, without producing dilatation to any extent; and, after giving a full opiate, have had the satisfaction of finding a marked improvement, after sufficient time had elapsed

* I will add, for the benefit of those interested in the discussion which some time since appeared in the columns of one of our medical journals, concerning the propriety of making forcible attempts to dilate the os with the fingers, that I have for several years practised it, and think that I have shortened the first stage of labor many times thereby. In cases where the anterior lip has not been fully dilated after expulsive pains began, I have also shortened the second stage, by persistently pressing upon it until it receded beyond the presenting portion of the head, even after it had become œdematous. The *rationale* is quite simple: the tissues being subjected to increased pressure, relax sooner than they otherwise would.

for its absorption, the patient having even harder contractions, with less distress than before, and the os uteri being speedily dilated.

During the first stage of a physiological labor, I believe that the circular fibres of the os are passively relaxed, and that the active, usually gentle, contraction of the fibres of the body of the uterus serves to overcome the resistance which the tissues of the cervix and os present to dilatation.

Now, if the circular fibres of the os retain their tonicity, or if they contract with those of the body during a pain (and I have felt them contracting in a number of cases), no dilatation can be effected; or, at any rate, it will be with great difficulty, and the resulting distress will be greater than when they offer only the minimum amount of resistance.

The cases above narrated and referred to have taught me that opium possesses the power of relaxing the circular fibres, at least of the os, and of stimulating the longitudinal and oblique fibres into active contraction. It is upon these principles that opium is exhibited in dysmenorrhœa, when it is dependent upon spasmodic contractions of the circular fibres; or where it is owing to the presence of "menstrual decidua," clots, etc.

In abortions it is an invaluable remedy, facilitating dilatation, diminishing hemorrhage, promoting the expulsion of the placenta, and lessening suffering.

Ergot, on the other hand, by causing contraction of the circular fibres, retains the placenta, and, therefore, should rarely be given (in abortion) until after the fœtus and secundines have been expelled.

*Placenta Prævia.*—I have used opium in three cases of placenta prævia, one at the sixth month, and two at term, saving the mother in each instance. In another case, I attempted to turn, but, having made an erroneous diagnosis as to position, the placenta being planted directly over the os uteri, I introduced the wrong hand, and, failing to get hold of the feet properly, so as to bring them down, I detached the (entire) placenta, rather than lose time by changing hands. I mistook a R. Occip. Post. for a L. O. Anter. position. The hæmorrhage ceased at once, and the mother subsequently did well.

I think that opium meets two important indications in *placenta prævia:* 1. It facilitates dilatation, thus shortening the period of greatest danger. 2. It promotes the expulsive power of the uterus. It also serves to lessen hemorrhage by a special hæmostatic action.

It is an interesting fact, that in one of these cases, when the respiration was reduced to four in the minute by cumulative action of the opium, which had been too frequently repeated by mistake, the uterus expelled the child with one pain, thus illustrating my statement that opium does not possess the power of arresting normal uterine contractions.

*Hour-Glass, Contractions, etc.*—While hour-glass, cylindrical, or other irregular tonic contractions of the uterus (particularly those which occur after the expulsion of the fœtus) may be (and doubtless are sometimes) spontaneous; still, in my experience, they have always seemed to be due to ergot. Since I have learned the special power of opium, as set forth in this paper, I have used it in these cases with invariable success, although some of the most approved obstetric authorities say such use "is objectionable."

I will give the following cases in point:—

CASE V. *December, 1864.* Mrs. McD., aged about 38. Primipara. Labor progressed steadily until the head had fully distended the perinæum. Retrocession followed every pain; and, as they were neither strong nor long no progress was made. The vulva, too, was well dilated, and I gave f ℥jss. Squibb's fl. ext. ergot to complete the delivery. Fifteen minutes afterward the peculiar contractions produced by ergot commenced, and the child was soon born. Placing my hand upon the fundus of the uterus (it having been pressed upon by the hand of an assistant while the child was being delivered, and the funis tied and severed), I discovered it to be much elongated, reaching above the umbilicus; and, making a vaginal examination, found the placenta to be beyond the reach of my fingers, and, introducing the hand, discovered it so tightly grasped by an hour-glass contraction that I could not remove it. Gave morph. sulph. gr. $\frac{1}{3}$, noting the time. A little less than half an hour afterward, I was awakened (having fallen asleep from great fatigue) by a contraction of the uterus under my hand. The placenta was expelled with considerable impulse, and the uterus contracted down almost entirely below the os pubis (which, by the way, judging by my experience, it seldom does, teachers and text-books to the contrary notwithstanding).

CASE VI. Mrs. S., multipara. A delayed labor dependent upon inefficient pains. Gave f. ʒjss. ergot. The child being born, I delivered the placenta at once (as I now invariably do after giving ergot). A cylindrical contraction immediately followed, the fundus rising considerably above the umbilicus, in fact almost as high as the ensiform cartilage. The cylinder was about three inches in diameter, firmly and uniformly contracted. An opiate was given, and in due time a permanent, globular contraction followed.

Dr. J. D. Trask, in his essay upon "Rupture of the Uterus," published in the *American Journal of Medical Sciences*, January and April, 1848, gives four cases of rupture of the uterus due to ergot. Malgaigne and others have reported similar cases. The following case is given to show how this accident *might* be produced in a diseased or even very powerful uterus, as well as to illustrate the apparently antagonistic effects of ergot and opium upon the gravid uterus.

Case VII. Mrs. M., multipara, about 35. Previous confinements easy. Present labor not worthy of note until the os was nearly obliterated, a ring only being left, when dilatation was for some unknown reason arrested, and no progress made for an hour. The uterine contractions then becoming inefficient, gave ʒjss. ergot. As soon as the pains peculiar to ergot began, I made an examination, and found the os less dilated than before, and its fibres contracting with those of the body. Gave gr. ⅓, morph. sulph. Within half an hour the pains had become more like those of a "physiological labor," the os uteri relaxed and became dilated, and the expulsion of the child speedily followed.

I gave opium in this case, with the expectation that it would produce a relaxation of the circular fibres of the os. It seems to have exerted this power in opposition to ergot as effectually as in the "hour-glass" and other irregular contractions above mentioned.

It may readily be seen that the simultaneous action of the ergot upon the os and body *might* have caused a rupture of the uterus. The contractions produced by ergot are continuous. I have often observed, however, that they have not been general, but have occurred in different sets of fibres successively. Herein lies one great danger of its use. In these, as well as all other irregular contractions of the uterus, I find opium a *prompt and reliable remedy.* In fact, I now use it in those cases of delayed labor dependent on inefficient uterine contractions, instead of ergot.

This property being established, the administration of opium admits of wide application in uterine therapeutics. In dysmenorrhœa, abortion, irregular contractions of the uterus of all kinds, previous, during, and subsequent to labor, and in placenta prævia as an adjuvant to Barnes' dilator, it will be found to be a valuable remedy; more certain in desired action (when given under proper indications) than any other remedy in our profession. Such at least it has been in my hands in quite an extensive obstetric experience.

In dysmenorrhœa, opium is given to quiet the contractions of the circular fibres (when this variety is present). In abortion, it is administered in the hope that the pains are caused by irregular contractions; and, if there is no dilatation (producing a partial separation of the membranes), it will often prove successful. If, on the contrary, the process has progressed so far as to render abortion inevitable, opium promotes it by relaxing the circular fibres of the os. It may appear at first strange to hear such apparently opposite properties ascribed to a remedy; but there is no inconsistency in the statement. The irregular (colicky) contractions do *not* constitute abortion, but they may produce it; and there is much less risk in a temporarily relaxed os uteri without pain, than in a normal condition of the os, with contractions of any kind in the body. In the "irregular contractions" at term (as in Cases V., VI., and VII.), it acts promptly,

I will state that cases might have been multiplied to a large number illustrating this subject; but the practical value of my paper would not have been enhanced at all, as the cases selected are as well or better-marked instances, each of its kind, than any others recorded by me since I commenced my observations seven years ago. (I had previously observed its powerlessness in quieting physiological uterine contractions, in the lying-in wards of Bellevue Hospital, while an interne.)

With respect to the mode of administration, I am not aware that it makes any difference what preparation of opium is used. I have generally employed solid opium pills, grs. 1 to 3, or morph. sulph., gr. $\frac{1}{6}$ to $\frac{1}{2}$ (the latter given dry on the tongue if nausea is present) at term. In abortion, if seen early, I give enemas of corresponding strength, usually employing from ʒss. to ʒj. doses of opii. tinct., in a tablespoonful of warm water, to be repeated after three hours if the pains continue, or later, if they return after being quieted. If abortion can not be prevented, the hypodermic syringe and Barnes' dilator of tampon are preferable. I have not used the hypodermic method at term, having been satisfied with the more gradual effects of administration by mouth; and, besides, I have thought that perhaps its rapid absorption into the maternal circulation *might* be injurious to the child. I have never observed any such effects following the administration by mouth. And, what is very singular, it seldom induces somnolency in the mother.

There are cases in which the os uteri (from previous inflammation) is almost, and sometimes entirely, undilatable, over which opium (in common with all other known therapeutic agents) exercises no control, and for which the knife is the remedy.

In conclusion, it will be seen that the theory set forth in this paper explains phenomena in the action of opium upon the gravid uterus, which have hitherto, from the time of Denman to the present, been regarded as exceptional, and that it establishes the value of this remedy as a parturient agent.

My object in calling the attention of the profession to these peculiar and very useful properties of opium has been the promotion of a wider knowledge, and a more general application of them in practice; and I feel assured, that if opium is administered in the conditions and doses above indicated, it would not disappoint the obstetric practitioner.—*New York Med. Journal.*

